# Anthropomorphic framing and failure comprehensibility influence different facets of trust towards industrial robots

**DOI:** 10.3389/frobt.2023.1235017

**Published:** 2023-09-07

**Authors:** Eileen Roesler

**Affiliations:** Department of Psychology, George Mason University, Fairfax, VA, United States

**Keywords:** human-robot interaction, trust, multi-dimensional trust, anthropomorphism, failure experience

## Abstract

**Introduction:** Utilizing anthropomorphic features in industrial robots is a prevalent strategy aimed at enhancing their perception as collaborative team partners and promoting increased tolerance for failures. Nevertheless, recent research highlights the presence of potential drawbacks associated with this approach. It is still widely unknown, how anthropomorphic framing influences the dynamics of trust especially, in context of different failure experiences.

**Method:** The current laboratory study wanted to close this research gap. To do so, fifty-one participants interacted with a robot that was either anthropomorphically or technically framed. In addition, each robot produced either a comprehensible or an incomprehensible failure.

**Results:** The analysis revealed no differences in general trust towards the technically and anthropomorphically framed robot. Nevertheless, the anthropomorphic robot was perceived as more transparent than the technical robot. Furthermore, the robot’s purpose was perceived as more positive after experiencing a comprehensible failure.

**Discussion:** The perceived higher transparency of anthropomorphically framed robots might be a double-edged sword, as the actual transparency did not differ between both conditions. In general, the results show that it is essential to consider trust multi-dimensionally, as a uni-dimensional approach which is often focused on performance might overshadow important facets of trust like transparency and purpose.

## 1 Introduction

Industrial robots are increasingly working hand in hand with their human coworkers. Hand in hand can be meant literally here, as close collaboration requires physical and temporal proximity (Onnasch and Roesler, 2021). For efficient collaboration, humans have to trust the robotic interaction partner (Hancock et al., 2011; Sheridan, 2016). While human-robot trust research is still an evolving field, trust has been studied extensively in human-automation and human-human interaction, both fields that are strongly related to human-robot interaction (HRI) (Lewis et al., 2018). Most theoretical models of trust in automation as well as trust in humans consider trust as multi-dimensional. For instance, for trust in automation, (Lee and See, 2004), performance, purpose, and process are described as separate dimensions of trust. Even though a transferability of these dimensions to human-robot trust is assumed (Lewis et al., 2018), recent research focused on using single-items of trust (e.g., Salem et al., 2015; Sarkar et al., 2017; Roesler et al., 2020; Onnasch and Hildebrandt, 2021) or uni-dimensional trust questionnaires (e.g., Sanders et al., 2019; Kopp et al., 2022). These approaches are not able to capture different dimensions, and thus cannot contribute much to a more detailed understanding of the underlying determinants of trust and trust dynamics in interaction with robots.

The multi-dimensional trust-in-automation questionnaire (MTQ) originally proposed by Wiczorek (2011) and translated, adapted, and validated by Roesler et al. (2022a) might also be used for investigating trust in HRI. Theoretically, it is based on the concept of Lee and See (2004) and assesses the dimensions performance, utility, purpose, and transparency. This allows for a more fine-grained assessment of trust in order to gain a better understanding of which trust dimensions are impacted from a given characteristic of a robot. Factors on part of the robot that influence trust can be classified as performance- and attribute-based characteristics (Hancock et al., 2011). In particular, performance-based factors such as reliability are the largest current influence on perceived trust in HRI. However, actual reliability is rarely correctly weighted for the formation of trust (Rieger et al., 2022). One decisive factor for this discrepancy could be the type of error experienced in the interaction (Madhavan et al., 2006). In particular, obvious failures made by a robot might dramatically reduce trust as expectations are violated (Madhavan et al., 2006). Based on this *easy-error hypothesis* in human-automation interaction, we hypothesized a comparable pattern in HRI. Thus, we assumed that comprehensible failures that might happen to humans as well are more forgivable than incomprehensible failures.

This effect could even be enhanced by one of the most popular design features in HRI—the application of anthropomorphic characteristics (Salem et al., 2015; Roesler et al., 2021). Anthropomorphism by design refers to the incorporation of human-like qualities and characteristics into the design and behavior of robots (Fischer, 2021). Anthropomorphic design extends beyond mere robotic appearances, encompassing elements such as communication, movement dynamics, and contextual integration (Onnasch and Roesler, 2021). Different factors collectively contribute to shaping perceived anthropomorphism of a robot. Even something subtle like an anthropomorphic framing of a robot can serve as a trigger that activates human-human interaction schemes (Onnasch and Roesler, 2019; Kopp et al., 2022). Due to the activation of humanlike expectations, failures that might have happened to a human as well [i.e., comprehensible failures (Madhavan et al., 2006)] could lead to less pronounced trust decrease in the anthropomorphically compared to the technically framed robot.

In addition to this presumed positive effect, anthropomorphism also comes with it potential pitfalls, especially in industrial HRI. In this application domain, anthropomorphism can undermine the perceived tool-like character of the robot, which can result in lower trust and perceived reliability (Roesler et al., 2020; Onnasch and Hildebrandt, 2021). The results in regard to anthropomorphic framing are currently mixed in task-related interactions (Onnasch and Roesler, 2019; Roesler et al., 2020; Kopp et al., 2022). Whereas studies which combined anthropomorphic framing and appearance in industrial HRI found negative effects (Onnasch and Roesler, 2019; Roesler et al., 2020), another study which investigated anthropomorphic framing without an exposure to an industrial robot found a positive effect on trust (Kopp et al., 2022). However, this was only the case if the anthropomorphic framing was combined with a cooperativeness framing (Kopp et al., 2022). As participants in this study were exposed to an actual robot and no additional framing in regard to the cooperativeness was given, it might be assumed that the possible mismatch of appearance, context, and framing reduces trust (Goetz et al., 2003; Roesler et al., 2022b). Thus, we hypothesized that anthropomorphic framing of an industrial robot leads to lower initial and learned trust compared to technical framing.

To investigate the joint effects of failure comprehensibility and anthropomorphic framing, we conducted a laboratory experiment. Participants collaborated with an industrial robot in a collaborative task. The robot either had an anthropomorphic framing or a technical framing based on perceived human-likeness framings used by Kopp et al. (2022). The dynamics of trust were investigated by measuring trust once initially before the actual collaboration started, after a period of perfectly reliable robotic performance, and after the experience of a failure, which was either comprehensible or incomprehensible.

## 2 Methods

The experiment was preregistered via the Open Science Framework (OSF) (https://osf.io/nvmqk) and approved by the local ethics committee. Also the collected data can be assessed via the OSF https://osf.io/2vzxj/.

### 2.1 Participants

The sample consisted of 51 participants (*M*_age_ = 26.94; *SD*_age_ = 7.72) who were recruited via the participant pool of the local university and online postings. Of those participants, 50.98% were female, 47.06% male, and 1.96% non-binary. Participants signed consent forms at the beginning of the experiment and received five Euros as compensation at the end of the experiment. Due to time constraints of the project, we were unable to achieve the intended sample size as planned and preregistered. Hence, it is crucial to consider the issue of limited statistical power.

### 2.2 Task and materials

**FIGURE 1:**
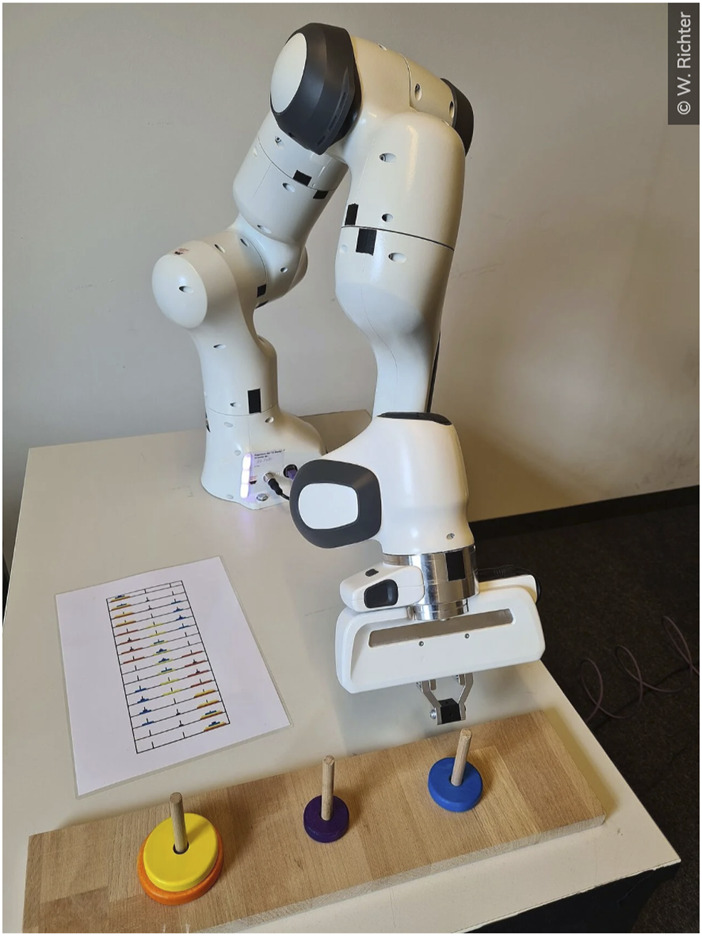
Photograph from a participant’s perspective of the shared human-robot workspace (© W. Richter received via https://www.tu.berlin/themen/campus-leben/roboter-mit-fehlern).

The aim of the human-robot collaboration was to solve multiple times a four-disk version of the Tower of Hanoi together with the industrial robot *Panda* (Figure 1). In this mathematical game, a stack of disks has to be moved from the leftmost to the rightmost peg by carrying only one disk at a time and never dragging a larger disk on a smaller one in the fewest possible moves. The tower was situated in front of the robot vis-à-vis the participant. The required movement sequences of the robot were preprogrammed and included movements in the following chronology. First, the robot moved toward one peg as a sign to remove the top disk from this peg. Subsequently, the robot moved toward another peg as a prompt to place the previously picked disk there. Afterward, the robot moved back to the resting position to start the next sequence. The participant’s task was to move the disks by following exactly the robot’s directives to solve the Tower of Hanoi in an optimal sequence. Moreover, the participant had the task to monitor the robot’s behavior by comparing the steps shown by the robot with an optimal procedure. The participants received a printed copy of the precise instructions of the Tower of Hanoi as can be seen on the table in Figure 1. Whenever the robot deviated from the optimal procedure, the participants needed to intervene by pushing a (mock-up) emergency button.

### 2.3 Dependent variables

Single items were used to assess general trust (How much do you trust the robot?) and reliability (How reliable is the robot?) both assessed on a scale from 0 to 100. In addition, the MTQ with four subscales (i.e, performance, utility, purpose, transparency) was assessed via 16 items (e.g., *The way the system works is clear to me.*) on a four-point Likert scale from *disagree* to *agree* (Wiczorek, 2011; Roesler et al., 2022a). Both the German and English versions of the questionnaire can be accessed through the OSF via https://osf.io/56cwx/.

To prevent confounding effects of participants’ interindividual differences we included two control variables. First, the disposition to trust technology was assessed (Lankton et al., 2015). Second, we asked participants to fill in a 5-item short version of the Interindividual Differences in Anthropomorphism Questionnaire Waytz et al. (2010). The short version comprised solely of items that directly addressed technological aspects (*To what extent does technology—devices and machines for manufacturing, entertainment, and productive processes (e.g., cars, computers, television sets)—have intentions?*).

To test whether the manipulation of anthropomorphism via framing was successful we incorporated a self-constructed questionnaire with ten items that addressed aspects of anthropomorphic context (e.g., the character, task, and preferences of the robot). All items were rated on a 0%–100% human-likeness scale. The manipulation of failure comprehensibility was checked by asking the participants to rate on a five-point Likert scale whether they too could have committed the failure (Roesler et al., 2020).

### 2.4 Procedure

All participants were randomly assigned to one of the four conditions and received corresponding written instructions including the framing of the robot. After filling out the initial questionnaire compromising single items of trust and perceived reliability, participants were informed that they will be working together with the robot for three blocks each including three Towers of Hanoi. After the first fault-free block, again the single items of trust and perceived reliability were assessed. The next block started and in the second block, either a comprehensible failure (i.e., showing the wrong position of a disc without the violation of rules) or an incomprehensible failure (i.e., showing the wrong position of a disc and breaking the rule of never putting a large disc on a smaller one) occurred. After the failure experience, participants needed to push the (mock-up) emergency button. This was done to ensure that all participants realized the failure. Subsequently, the single items of trust and perceived reliability, the MTQ, sociodemographics, control variables, and manipulation checks were measured. After this, all participants were debriefed and obtained the 5 Euro compensation. The entire experiment lasted approximately 35 min.

### 2.5 Design

The study consisted of a 2 × 2 × 3 mixed design with the two between-factors robots framing (anthropomorphic vs technical) and failure comprehensibility (low vs high) and the within-factor experience (initial vs pre failure vs post failure).

The different robot framing conditions were implemented via written instructions (Kopp et al., 2022). In the anthropomorphic conditions, the robot was framed as a colleague and named Paul with humanlike characteristics. In contrast, in the technical conditions, the framing characterized the robot as a tool with some technical specifications and the model name PR-5. The framings can also be accessed via the OSF (https://osf.io/3xgcp). The failures were represented by wrong instructions on part of the robot. The comprehensibility was manipulated by the obviousness of the failure. In incomprehensible conditions, the robot suggested moving a bigger disk on a smaller one, which is forbidden by the general rules of the Tower of Hanoi. In the comprehensible conditions, the robot suggested a wrong position of a disk without breaking a general rule.

## 3 Results

### 3.1 Control variables

First, the variables regarding the individual differences concerning attitudes toward technology and tendency to anthropomorphize were analyzed between the four conditions using one-way ANOVAs. The analyses revealed no significant differences between the four groups in the disposition to trust technology (*F*(3, 47) = 1.25; *p* = .303), as well as the tendency to anthropomorphize (*F*(3, 47) = 2.48; *p* = .072).

### 3.2 Manipulation check

To investigate whether the manipulations were successful, independent t-tests were conducted. Surprisingly, the anthropomorphically framed robot was not perceived as significantly more anthropomorphic on the self-constructed scale compared to the technically framed one (*t*(49) = 0.34; *p* = .732). Moreover, the comprehensible and incomprehensible failures did not lead to a different understandability of the failure (*t*(49) = −0.96; *p* = .341).

### 3.3 Initial trust

Initial trust and perceived reliability were analyzed in regard to differences between differently framed robots via independent t-tests. The analyses revealed neither a difference in general trust (*t*(49) = −0.63; *p* = .529) nor in perceived reliability (*t*(49) = 1.48; *p* = .145) between the framing conditions.

### 3.4 Learned trust

General trust and perceived reliability were analyzed via 2 × 2 × 2 mixed ANOVAs with the between-factors framing (anthropomorphic vs technical) and failure comprehensibility (low vs high) as well as the within-factor failure experience (pre-vs. post-failure). The analysis of trust revealed only a significant main effect of failure experience (*F*(1, 47) = 40.73; *p* < .001) with higher trust before (*M* = 84.75; *SD* = 17.90) compared to after the failure experience (*M* = 64.31; *SD* = 24.65). No further main or interaction effects were revealed in the analysis (all *ps* > .068). A comparable pattern of results was revealed for perceived reliability. Again, a significant main effect of failure experience was found (*F*(1, 47) = 71.15; *p* < .001). Participants perceived the robot prior failure experience (*M* = 93.51; *SD* = 8.94) as significantly more reliable than after failure experience (*M* = 66.16; *SD* = 23.65). No further effects were revealed (all *ps* > .349).

**FIGURE 2:**
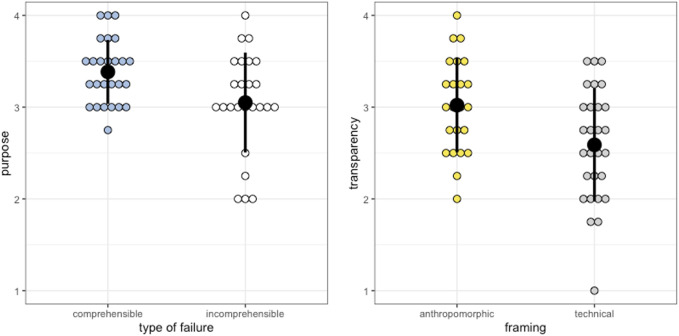
Means, standard errors and exact values of each participant for the type of failure concerning purpose (left) and the framing concerning transparency (right).

As the MTQ was measured after failure experience 2 × 2 between-factors ANOVAs with the factors framing (anthropomorphic vs technical) and failure comprehensibility (low vs high) were used. Neither the analysis of the performance scale nor the analysis of the utility scale revealed any significant effects (all *ps* > .132). However, the analysis of the purpose scale showed a significant main effect of failure comprehensibility (*F*(1, 47) = 6.20; *p* = .016) depicted in Figure 2 (left). Incomprehensible failures (*M* = 3.05; *SD* = 0.54) received significantly lower scores on this scale compared to comprehensible failures (*M* = 3.38; *SD* = 0.35). Moreover, the analysis of the transparency scale revealed a significant main effect of robot framing (*F*(1, 47) = 7.08; *p* = .011) as can be seen in Figure 2 (right). The anthropomorphically framed robot (*M* = 3.02; *SD* = 0.52) was perceived as significantly more transparent than the technically framed one (*M* = 2.59; *SD* = 0.62). No further significant effects were revealed for the purpose and transparency scale (all *ps* > .161).

## 4 Discussion

The purpose of the presented study was to examine the joint effects of anthropomorphic robot framing and the experience of more or less comprehensible failures on human trust in a realistic industrial human-robot collaboration. Based on previous research in task-related HRI (Onnasch and Roesler, 2019; Roesler et al., 2020; Onnasch and Hildebrandt, 2021) it was assumed that anthropomorphic framing would lead to lower trust and perceived reliability compared to a technical framing. The present results were not consistent with this claim, as no significant differences in initial and learned trust as well as perceived reliability were revealed. This might be explained by the interplay of framing and appearance. Earlier studies in industrial HRI manipulated framing and appearance together (Roesler et al., 2020; Onnasch and Hildebrandt, 2021). The comparison to the current results could indicate that the negative effect of the decorative anthropomorphism in industrial HRI might be mainly attributable to appearance rather than to framing. In addition, recent research of Kopp et al. (2022) showed a positive effect of anthropomorphic framing on trust in industrial HRI if the relation is perceived as cooperative. Even though it often remains unclear if and why people perceive the relation to an industrial robot in a cooperative or competitive manner (Oliveira et al., 2018), our interaction scenario was designed in a cooperative way. This might explain why anthropomorphic framing was influencing at least one facet of trust—transparency.

As anthropomorphism is assumed to activate well-known human-human interaction scripts, knowledge about the otherwise highly unknown novel technology is elicited (Epley et al., 2007). The imputation of human-like functions and behaviors can thus reduce uncertainty and, in this case, increase perceived transparency. Of course, this is a double-edged sword, as perceived transparency does not refer to actual transparency in this case. The illusion of higher transparency might even lead to unintentional side effects, such as a wrong mental model of the robot. In terms of future research, it would be important to consolidate the current findings by further examining the effect of anthropomorphic framing on transparency. However, the general effectiveness of framing in regard to human-robot trust should be interpreted with caution as no significant results were revealed for general trust and the other subscales of the MTQ. This pattern of results is consistent with a current meta-analysis showing no significant effect of context anthropomorphism for subjective as well as objective outcomes (Roesler et al., 2021). However, the meta-analysis has shed light on a notable research gap concerning anthropomorphic context, which has received comparably less attention than studying the effectiveness of robot appearances. The findings of this study, coupled with insights from Kopp et al. (2022) ’s previous work, tentatively suggest a potential effectiveness of anthropomorphic framing for industrial HRI in regard to trust. The previous and current results underscore the necessity for further exploration and empirical investigation of possible benefits of anthropomorphic framing in industrial HRI.

Therefore, it might be not surprising that3no interaction effect of framing and failure comprehensibility was found. The possible effect might have been covered by the rather non-salient manipulations of both anthropomorphism and failure comprehensibility. This assumption is further supported by the non-significant manipulation checks for both variables. Nonetheless, the comprehensibility of failures did significantly influence the perceived purpose of the robot. Purpose refers to motives, benevolence, and intentions (Lee and See, 2004) and not to the performance of the interaction partner. This leads to the assumption that failure number and types affect different facets of trust.

Both the result that anthropomorphic framing and failure comprehensibility can affect different dimensions of trust but not general trust shows the importance to integrate multi-dimensional approaches to investigate trust in HRI. Uni-dimensional trust measures most commonly relate to performance aspects (Roesler et al., 2022b). Even though performance-attributes of a robot are one of the most important determinants of trust, they are by far not the only one (Hancock et al., 2011). Therefore, it is highly relevant to also include trust facets that go beyond performance. Thus, future research should include a multi-dimensional view at trust, particularly with novel embodied technologies like robots.

Although the generality of the current results must be established by future research, especially with bigger samples sizes to investigate the joint effect of both factors, the present study has provided clear support that uni-dimensional trust measurements might overshadow certain important facets of trust. Not only was anthropomorphic framing leading to higher transparency compared to technical framing, but more comprehensible failures to more perceived purpose of the robot compared to incomprehensible failures. Furthermore, this research opens up multiple avenues for future research to investigate more detailed different dimensions of trust.

## Data Availability

The datasets presented in this study can be found in online repositories. The names of the repository/repositories and accession number(s) can be found below: https://osf.io/2vzxj/.
